# 10-[2-(Dimethyl­amino)eth­yl]-9-(4-methoxy­phen­yl)-3,3,6,6-tetra­methyl-3,4,6,7,9,10-hexa­hydro­acridine-1,8(2*H*,5*H*)-dione

**DOI:** 10.1107/S1600536808043882

**Published:** 2009-01-10

**Authors:** P. Balamurugan, R. Jagan, V. Thiagarajan, Bohari M. Yamin, K. Sivakumar

**Affiliations:** a Department of Physics, Dhanalakshmi College of Engineering, Tambaram, Chennai 601 301, India; b Department of Physics, Anna University, Chennai 600 025, India; c National Centre for Ultrafast Processes, University of Madras, Taramani Campus, Chennai 600 113, India; d School of Chemical Sciences and Food Technology, Faculty of Science and Technology, Universiti Kebangsaan Malaysia, UKM 43600, Bangi Selangor, Malaysia

## Abstract

In the title compound, C_28_H_38_N_2_O_3_, the central ring of the acridinedione system adopts a boat conformation, while one of the outer rings adopts a half-chair conformation and the conformation of the other outer ring is between a sofa and a half-chair. The acridinedione system is buckled, with an angle of 22.01 (3)°. The crystal packing comprises layers of mol­ecules laid parallel to the *ac* plane, being reinforced by an intermolecular C—H⋯O interaction.

## Related literature

For related literature, see: Josephrajan *et al.* (2005 ▶); Murugan *et al.* (1998 ▶); Srividya *et al.* (1996 ▶, 1998 ▶); Nardelli (1983 ▶).
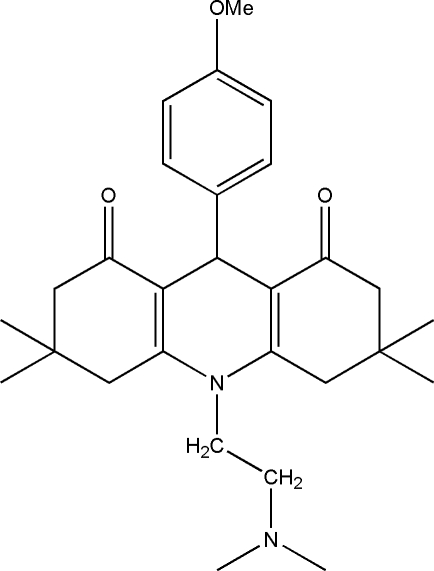


## Experimental

### 

#### Crystal data

C_28_H_38_N_2_O_3_
*M* *_r_* = 450.60
Monoclinic, 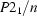

*a* = 10.3030 (13) Å
*b* = 19.299 (3) Å
*c* = 13.3961 (18) Å
β = 103.336 (4)°
*V* = 2591.8 (6) Å^3^
*Z* = 4
Mo *K*α radiation
μ = 0.07 mm^−1^
*T* = 295 (2) K
0.56 × 0.16 × 0.10 mm

#### Data collection

Bruker KappaAPEXII CCD diffractometer
Absorption correction: multi-scan (*SADABS*; Sheldrick, 2004 ▶) *T*_min_ = 0.95, *T*_max_ = 0.99
17538 measured reflections
5944 independent reflections
3567 reflections with *I* > 2σ(*I*)
*R*_int_ = 0.041

#### Refinement

*R*[*F* ^2^ > 2σ(*F* ^2^)] = 0.064
*wR*(*F* ^2^) = 0.162
*S* = 1.01
5944 reflections
298 parameters
H-atom parameters constrained
Δρ_max_ = 0.23 e Å^−3^
Δρ_min_ = −0.16 e Å^−3^

### 

Data collection: *APEX2* (Bruker, 2004 ▶); cell refinement: *APEX2* and *SAINT* (Bruker, 2004 ▶); data reduction: *SAINT* and *XPREP* (Bruker, 2004 ▶); program(s) used to solve structure: *SHELXS97* (Sheldrick, 2008 ▶); program(s) used to refine structure: *SHELXL97* (Sheldrick, 2008 ▶); molecular graphics: *ORTEP-3* (Farrugia, 1997 ▶) and *PLATON* (Spek, 2003 ▶); software used to prepare material for publication: *SHELXL97*.

## Supplementary Material

Crystal structure: contains datablocks I, global. DOI: 10.1107/S1600536808043882/tk2350sup1.cif

Structure factors: contains datablocks I. DOI: 10.1107/S1600536808043882/tk2350Isup2.hkl

Additional supplementary materials:  crystallographic information; 3D view; checkCIF report

## Figures and Tables

**Table 1:** Hydrogen-bond geometry (Å, °)

*D*—H⋯*A*	*D*—H	H⋯*A*	*D*⋯*A*	*D*—H⋯*A*
C14—H14*B*⋯O1^i^	0.97	2.51	3.368 (2)	147

Symmetry code: (i) 
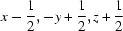
.

**Fig. 1.**
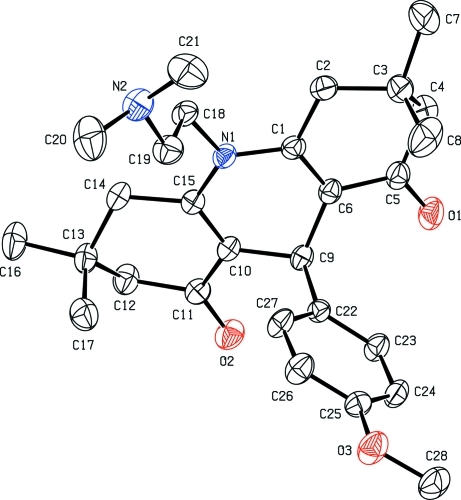
The molecular structure of (I), showing 30% probability displacement ellipsoids. H atoms have been omitted for clarity.

## supplementary crystallographic information

### Comment

Acridines, the earliest known antibiotics, are toxic towards bacteria. Some acridinedione derivatives show good inhibition against the pathogen *Vibrio* isolate-I (Josephrajan *et al.*, 2005). Certain acridine-1,8-diones exhibit fluorescence activities (Murugan *et al.*, 1998) and a few acridinedione derivatives also show photophysical (Srividya *et al.*, 1998) and electrochemical properties (Srividya *et al.*, 1996). Thus, the accurate description of crystal structures of substituted acridinediones are expected to provide useful information on the role of substituents in influencing molecular conformation which has a direct relationship to biological activity. This paper deals with the precise description of a 4-methoxyphenyl substituted tetramethyl acridinedione, (I).

The planar phenyl ring of the substituent moiety at C9 is perpendicular to the acridinedione moiety forming a dihedral angle of 88.21 (6)°, Fig. 1. The dimethylaminoethyl group is also oriented 80.0 (1)° to the acridinedione plane. The substitutuents at the C9 and N1 positions are *cis* oriented with respect to the acridinedione moiety and project opposite to the fold in the acridinedione moiety. The central ring of the acridinedione moiety adopts a boat conformation (ΔC_s_ (N1) = 0.028 (1)° & ΔC_s_ (C6—C1) = 0.057 (1)°). One of the outer rings (C1—C6) adopts a half-chair conformation (ΔC_2_ (C1—C6) = 0.045 (1)°) and that of the other outer ring (C10—C15) ring is between a sofa and half chair conformation (ΔC_s_ (C10) = 0.066 (1)° & ΔC_2_ (C10—C15) = 0.061 (1)°) (Nardelli, 1983). The crystal packing consists of layers of molecules laid parallel to the *ac*-plane. Only one of the two keto-O atoms participates in a C—H···O contact, Table 1.

### Experimental

Light-yellow crystals were obtained by recrystallization from an ethanol solution of (I).

### Refinement

H atoms were placed in geometrically idealized positions and allowed to ride on their parent atoms, with C – H distances in the range 0.93 – 0.98 Å, and with *U*_iso_(H) = 1.2 or 1.5 (for methyl-H) times *U*_eq_(C).

### Crystal data

**Table:**

C_28_H_38_N_2_O_3_	*F*(000) = 976
*M**_r_* = 450.60	*D*_x_ = 1.155 Mg m^−^^3^
Monoclinic, *P*2_1_/*n*	Mo *K*α radiation, λ = 0.71073 Å
Hall symbol: -P 2yn	Cell parameters from 2202 reflections
*a* = 10.3030 (13) Å	θ = 1.9–27.5°
*b* = 19.299 (3) Å	µ = 0.07 mm^−^^1^
*c* = 13.3961 (18) Å	*T* = 295 K
β = 103.336 (4)°	Slab, light yellow
*V* = 2591.8 (6) Å^3^	0.56 × 0.16 × 0.10 mm
*Z* = 4	

### Data collection

**Table:**

Bruker KappaAPEXII CCD diffractometer	5944 independent reflections
Radiation source: fine-focus sealed tube	3567 reflections with *I* > 2σ(*I*)
graphite	*R*_int_ = 0.041
ω and φ scans	θ_max_ = 27.6°, θ_min_ = 1.9°
Absorption correction: multi-scan (*SADABS*; Sheldrick, 2004)	*h* = −9→13
*T*_min_ = 0.95, *T*_max_ = 0.99	*k* = −23→25
17538 measured reflections	*l* = −17→17

### Refinement

**Table:**

Refinement on *F*^2^	Primary atom site location: structure-invariant direct methods
Least-squares matrix: full	Secondary atom site location: difference Fourier map
*R*[*F*^2^ > 2σ(*F*^2^)] = 0.064	Hydrogen site location: inferred from neighbouring sites
*wR*(*F*^2^) = 0.162	H-atom parameters constrained
*S* = 1.01	*w* = 1/[σ^2^(*F*_o_^2^) + (0.0679*P*)^2^ + 0.3097*P*] where *P* = (*F*_o_^2^ + 2*F*_c_^2^)/3
5944 reflections	(Δ/σ)_max_ < 0.001
298 parameters	Δρ_max_ = 0.23 e Å^−^^3^
0 restraints	Δρ_min_ = −0.16 e Å^−^^3^

### Special details

**Table:**

Geometry. All e.s.d.'s (except the e.s.d. in the dihedral angle between two l.s. planes) are estimated using the full covariance matrix. The cell e.s.d.'s are taken into account individually in the estimation of e.s.d.'s in distances, angles and torsion angles; correlations between e.s.d.'s in cell parameters are only used when they are defined by crystal symmetry. An approximate (isotropic) treatment of cell e.s.d.'s is used for estimating e.s.d.'s involving l.s. planes.
Refinement. Refinement of *F*^2^ against ALL reflections. The weighted *R*-factor *wR* and goodness of fit *S* are based on *F*^2^, conventional *R*-factors *R* are based on *F*, with *F* set to zero for negative *F*^2^. The threshold expression of *F*^2^ > σ(*F*^2^) is used only for calculating *R*-factors(gt) *etc*. and is not relevant to the choice of reflections for refinement. *R*-factors based on *F*^2^ are statistically about twice as large as those based on *F*, and *R*- factors based on ALL data will be even larger.

### Fractional atomic coordinates and isotropic or equivalent isotropic displacement parameters (Å^2^)

**Table:**

	*x*	*y*	*z*	*U*_iso_*/*U*_eq_	
O1	0.88988 (15)	0.24189 (8)	0.17052 (12)	0.0631 (5)	
O2	0.50984 (16)	0.38481 (8)	0.19363 (11)	0.0601 (4)	
O3	0.32638 (16)	0.06075 (9)	0.00092 (12)	0.0715 (5)	
N1	0.69322 (16)	0.22382 (8)	0.45422 (12)	0.0417 (4)	
N2	0.5859 (2)	0.10112 (10)	0.63649 (14)	0.0637 (5)	
C1	0.78666 (19)	0.20226 (10)	0.39984 (15)	0.0410 (5)	
C2	0.8942 (2)	0.15337 (11)	0.45263 (16)	0.0528 (6)	
H2A	0.8534	0.1155	0.4819	0.063*	
H2B	0.9530	0.1777	0.5087	0.063*	
C3	0.9777 (2)	0.12330 (13)	0.38223 (18)	0.0655 (7)	
C4	1.0128 (2)	0.18105 (14)	0.31611 (18)	0.0680 (7)	
H4A	1.0704	0.2141	0.3597	0.082*	
H4B	1.0621	0.1617	0.2691	0.082*	
C5	0.8921 (2)	0.21803 (11)	0.25587 (17)	0.0486 (5)	
C6	0.78033 (19)	0.22640 (10)	0.30388 (14)	0.0404 (5)	
C7	1.1063 (3)	0.0932 (2)	0.4501 (2)	0.1156 (13)	
H7A	1.1604	0.0739	0.4075	0.173*	
H7B	1.0840	0.0576	0.4933	0.173*	
H7C	1.1549	0.1294	0.4920	0.173*	
C8	0.8996 (3)	0.06640 (14)	0.3143 (2)	0.0950 (10)	
H8A	0.9527	0.0481	0.2702	0.143*	
H8B	0.8186	0.0854	0.2734	0.143*	
H8C	0.8786	0.0299	0.3567	0.143*	
C9	0.65777 (19)	0.26291 (9)	0.24426 (14)	0.0394 (5)	
H9	0.6859	0.2967	0.1988	0.047*	
C10	0.59692 (19)	0.30198 (10)	0.31887 (14)	0.0384 (4)	
C11	0.5246 (2)	0.36554 (10)	0.28247 (15)	0.0436 (5)	
C12	0.4725 (2)	0.40739 (11)	0.35877 (16)	0.0549 (6)	
H12A	0.3988	0.4358	0.3227	0.066*	
H12B	0.5422	0.4382	0.3946	0.066*	
C13	0.4252 (2)	0.36234 (11)	0.43689 (16)	0.0506 (5)	
C14	0.5418 (2)	0.31647 (10)	0.48961 (15)	0.0470 (5)	
H14A	0.6068	0.3449	0.5359	0.056*	
H14B	0.5093	0.2820	0.5306	0.056*	
C15	0.61041 (19)	0.27989 (9)	0.41677 (14)	0.0389 (4)	
C16	0.3845 (3)	0.40841 (12)	0.51759 (19)	0.0737 (8)	
H16A	0.3542	0.3799	0.5664	0.111*	
H16B	0.3140	0.4389	0.4847	0.111*	
H16C	0.4599	0.4353	0.5524	0.111*	
C17	0.3062 (2)	0.31810 (13)	0.3845 (2)	0.0692 (7)	
H17A	0.3313	0.2889	0.3341	0.104*	
H17B	0.2340	0.3477	0.3516	0.104*	
H17C	0.2783	0.2899	0.4347	0.104*	
C18	0.6893 (2)	0.19211 (11)	0.55353 (15)	0.0484 (5)	
H18A	0.6770	0.2280	0.6011	0.058*	
H18B	0.7737	0.1693	0.5818	0.058*	
C19	0.5771 (2)	0.13980 (12)	0.54196 (17)	0.0599 (6)	
H19A	0.4921	0.1637	0.5239	0.072*	
H19B	0.5815	0.1079	0.4869	0.072*	
C20	0.4552 (3)	0.08163 (17)	0.6512 (2)	0.1008 (11)	
H20A	0.4654	0.0565	0.7144	0.151*	
H20B	0.4108	0.0529	0.5952	0.151*	
H20C	0.4032	0.1226	0.6537	0.151*	
C21	0.6703 (3)	0.04104 (14)	0.6416 (2)	0.0922 (9)	
H21A	0.6759	0.0175	0.7056	0.138*	
H21B	0.7579	0.0553	0.6366	0.138*	
H21C	0.6334	0.0103	0.5859	0.138*	
C22	0.56405 (19)	0.21121 (9)	0.17768 (14)	0.0389 (4)	
C23	0.5755 (2)	0.19818 (11)	0.07903 (15)	0.0497 (5)	
H23	0.6375	0.2232	0.0531	0.060*	
C24	0.4982 (2)	0.14919 (11)	0.01715 (16)	0.0544 (6)	
H24	0.5080	0.1420	−0.0494	0.065*	
C25	0.4071 (2)	0.11125 (11)	0.05418 (16)	0.0495 (5)	
C26	0.3929 (2)	0.12379 (12)	0.15253 (17)	0.0595 (6)	
H26	0.3306	0.0988	0.1782	0.071*	
C27	0.4700 (2)	0.17284 (11)	0.21265 (16)	0.0529 (6)	
H27	0.4589	0.1805	0.2787	0.063*	
C28	0.3427 (3)	0.04469 (14)	−0.09916 (19)	0.0768 (8)	
H28A	0.2812	0.0088	−0.1286	0.115*	
H28B	0.4323	0.0291	−0.0949	0.115*	
H28C	0.3256	0.0853	−0.1415	0.115*	

### Atomic displacement parameters (Å^2^)

**Table:**

	*U*^11^	*U*^22^	*U*^33^	*U*^12^	*U*^13^	*U*^23^
O1	0.0671 (11)	0.0764 (11)	0.0546 (10)	−0.0024 (8)	0.0320 (8)	0.0013 (8)
O2	0.0815 (12)	0.0558 (9)	0.0444 (9)	0.0103 (8)	0.0171 (8)	0.0126 (7)
O3	0.0749 (11)	0.0787 (12)	0.0624 (11)	−0.0261 (9)	0.0192 (9)	−0.0271 (9)
N1	0.0492 (10)	0.0449 (9)	0.0323 (8)	0.0027 (8)	0.0120 (8)	0.0030 (7)
N2	0.0791 (14)	0.0595 (12)	0.0561 (12)	−0.0048 (10)	0.0231 (11)	0.0131 (10)
C1	0.0421 (11)	0.0415 (11)	0.0379 (11)	−0.0038 (8)	0.0065 (9)	−0.0051 (9)
C2	0.0551 (14)	0.0569 (13)	0.0424 (12)	0.0084 (11)	0.0031 (10)	−0.0024 (10)
C3	0.0672 (16)	0.0759 (17)	0.0521 (14)	0.0281 (13)	0.0111 (12)	−0.0006 (13)
C4	0.0499 (14)	0.097 (2)	0.0589 (15)	0.0114 (13)	0.0163 (12)	−0.0108 (14)
C5	0.0497 (13)	0.0510 (13)	0.0477 (13)	−0.0080 (10)	0.0167 (10)	−0.0124 (10)
C6	0.0443 (11)	0.0391 (11)	0.0388 (11)	−0.0044 (8)	0.0115 (9)	−0.0060 (8)
C7	0.099 (2)	0.165 (3)	0.084 (2)	0.078 (2)	0.0213 (19)	0.014 (2)
C8	0.152 (3)	0.0593 (17)	0.0743 (19)	0.0223 (18)	0.027 (2)	−0.0124 (15)
C9	0.0482 (12)	0.0392 (10)	0.0335 (10)	−0.0023 (9)	0.0147 (9)	0.0032 (8)
C10	0.0462 (11)	0.0364 (10)	0.0339 (10)	−0.0032 (8)	0.0117 (9)	−0.0009 (8)
C11	0.0496 (12)	0.0409 (11)	0.0407 (11)	−0.0041 (9)	0.0115 (10)	0.0018 (9)
C12	0.0716 (15)	0.0449 (12)	0.0517 (13)	0.0103 (11)	0.0212 (12)	0.0036 (10)
C13	0.0637 (14)	0.0461 (12)	0.0474 (12)	0.0084 (10)	0.0238 (11)	−0.0018 (10)
C14	0.0610 (14)	0.0462 (12)	0.0366 (11)	−0.0033 (10)	0.0172 (10)	−0.0052 (9)
C15	0.0426 (11)	0.0368 (11)	0.0381 (11)	−0.0044 (8)	0.0110 (9)	−0.0015 (8)
C16	0.105 (2)	0.0610 (15)	0.0668 (17)	0.0224 (14)	0.0435 (16)	0.0001 (13)
C17	0.0590 (16)	0.0802 (18)	0.0734 (17)	−0.0012 (13)	0.0257 (13)	−0.0027 (14)
C18	0.0582 (13)	0.0523 (13)	0.0350 (11)	0.0042 (10)	0.0116 (10)	0.0080 (9)
C19	0.0642 (15)	0.0624 (15)	0.0540 (14)	−0.0022 (12)	0.0153 (12)	0.0129 (11)
C20	0.109 (2)	0.103 (2)	0.111 (3)	−0.0014 (19)	0.066 (2)	0.021 (2)
C21	0.094 (2)	0.077 (2)	0.102 (2)	0.0129 (17)	0.0145 (18)	0.0305 (17)
C22	0.0430 (11)	0.0408 (11)	0.0327 (10)	0.0047 (8)	0.0081 (9)	0.0014 (8)
C23	0.0547 (13)	0.0582 (13)	0.0395 (12)	−0.0091 (10)	0.0175 (10)	−0.0032 (10)
C24	0.0597 (14)	0.0679 (15)	0.0378 (12)	−0.0049 (12)	0.0156 (11)	−0.0132 (11)
C25	0.0496 (13)	0.0505 (12)	0.0465 (12)	−0.0045 (10)	0.0073 (10)	−0.0083 (10)
C26	0.0674 (16)	0.0640 (15)	0.0511 (13)	−0.0199 (12)	0.0221 (12)	−0.0038 (11)
C27	0.0676 (15)	0.0588 (14)	0.0356 (11)	−0.0114 (11)	0.0189 (11)	−0.0028 (10)
C28	0.0816 (19)	0.0837 (19)	0.0629 (16)	−0.0160 (15)	0.0124 (14)	−0.0311 (14)

### Geometric parameters (Å, °)

**Table:**

O1—C5	1.228 (2)	C12—H12B	0.9700
O2—C11	1.222 (2)	C13—C17	1.526 (3)
O3—C25	1.370 (2)	C13—C14	1.528 (3)
O3—C28	1.423 (3)	C13—C16	1.531 (3)
N1—C15	1.398 (2)	C14—C15	1.506 (2)
N1—C1	1.398 (2)	C14—H14A	0.9700
N1—C18	1.473 (2)	C14—H14B	0.9700
N2—C21	1.442 (3)	C16—H16A	0.9600
N2—C20	1.454 (3)	C16—H16B	0.9600
N2—C19	1.455 (3)	C16—H16C	0.9600
C1—C6	1.355 (3)	C17—H17A	0.9600
C1—C2	1.502 (3)	C17—H17B	0.9600
C2—C3	1.529 (3)	C17—H17C	0.9600
C2—H2A	0.9700	C18—C19	1.515 (3)
C2—H2B	0.9700	C18—H18A	0.9700
C3—C4	1.518 (3)	C18—H18B	0.9700
C3—C8	1.531 (4)	C19—H19A	0.9700
C3—C7	1.538 (3)	C19—H19B	0.9700
C4—C5	1.498 (3)	C20—H20A	0.9600
C4—H4A	0.9700	C20—H20B	0.9600
C4—H4B	0.9700	C20—H20C	0.9600
C5—C6	1.452 (3)	C21—H21A	0.9600
C6—C9	1.505 (3)	C21—H21B	0.9600
C7—H7A	0.9600	C21—H21C	0.9600
C7—H7B	0.9600	C22—C23	1.377 (2)
C7—H7C	0.9600	C22—C27	1.384 (3)
C8—H8A	0.9600	C23—C24	1.382 (3)
C8—H8B	0.9600	C23—H23	0.9300
C8—H8C	0.9600	C24—C25	1.370 (3)
C9—C10	1.500 (2)	C24—H24	0.9300
C9—C22	1.525 (3)	C25—C26	1.380 (3)
C9—H9	0.9800	C26—C27	1.372 (3)
C10—C15	1.355 (2)	C26—H26	0.9300
C10—C11	1.459 (3)	C27—H27	0.9300
C11—C12	1.496 (3)	C28—H28A	0.9600
C12—C13	1.524 (3)	C28—H28B	0.9600
C12—H12A	0.9700	C28—H28C	0.9600
			
C25—O3—C28	117.13 (18)	C15—C14—C13	114.11 (16)
C15—N1—C1	118.69 (15)	C15—C14—H14A	108.7
C15—N1—C18	120.27 (15)	C13—C14—H14A	108.7
C1—N1—C18	120.84 (16)	C15—C14—H14B	108.7
C21—N2—C20	110.7 (2)	C13—C14—H14B	108.7
C21—N2—C19	111.7 (2)	H14A—C14—H14B	107.6
C20—N2—C19	112.2 (2)	C10—C15—N1	120.58 (16)
C6—C1—N1	120.26 (18)	C10—C15—C14	121.39 (17)
C6—C1—C2	122.10 (17)	N1—C15—C14	117.98 (16)
N1—C1—C2	117.62 (16)	C13—C16—H16A	109.5
C1—C2—C3	114.00 (17)	C13—C16—H16B	109.5
C1—C2—H2A	108.8	H16A—C16—H16B	109.5
C3—C2—H2A	108.8	C13—C16—H16C	109.5
C1—C2—H2B	108.8	H16A—C16—H16C	109.5
C3—C2—H2B	108.8	H16B—C16—H16C	109.5
H2A—C2—H2B	107.6	C13—C17—H17A	109.5
C4—C3—C2	108.96 (19)	C13—C17—H17B	109.5
C4—C3—C8	110.1 (2)	H17A—C17—H17B	109.5
C2—C3—C8	110.2 (2)	C13—C17—H17C	109.5
C4—C3—C7	109.5 (2)	H17A—C17—H17C	109.5
C2—C3—C7	107.98 (19)	H17B—C17—H17C	109.5
C8—C3—C7	110.1 (2)	N1—C18—C19	111.32 (17)
C5—C4—C3	112.6 (2)	N1—C18—H18A	109.4
C5—C4—H4A	109.1	C19—C18—H18A	109.4
C3—C4—H4A	109.1	N1—C18—H18B	109.4
C5—C4—H4B	109.1	C19—C18—H18B	109.4
C3—C4—H4B	109.1	H18A—C18—H18B	108.0
H4A—C4—H4B	107.8	N2—C19—C18	111.11 (18)
O1—C5—C6	121.6 (2)	N2—C19—H19A	109.4
O1—C5—C4	121.02 (19)	C18—C19—H19A	109.4
C6—C5—C4	117.34 (19)	N2—C19—H19B	109.4
C1—C6—C5	120.83 (19)	C18—C19—H19B	109.4
C1—C6—C9	121.04 (17)	H19A—C19—H19B	108.0
C5—C6—C9	118.13 (17)	N2—C20—H20A	109.5
C3—C7—H7A	109.5	N2—C20—H20B	109.5
C3—C7—H7B	109.5	H20A—C20—H20B	109.5
H7A—C7—H7B	109.5	N2—C20—H20C	109.5
C3—C7—H7C	109.5	H20A—C20—H20C	109.5
H7A—C7—H7C	109.5	H20B—C20—H20C	109.5
H7B—C7—H7C	109.5	N2—C21—H21A	109.5
C3—C8—H8A	109.5	N2—C21—H21B	109.5
C3—C8—H8B	109.5	H21A—C21—H21B	109.5
H8A—C8—H8B	109.5	N2—C21—H21C	109.5
C3—C8—H8C	109.5	H21A—C21—H21C	109.5
H8A—C8—H8C	109.5	H21B—C21—H21C	109.5
H8B—C8—H8C	109.5	C23—C22—C27	116.67 (18)
C10—C9—C6	108.08 (15)	C23—C22—C9	119.86 (17)
C10—C9—C22	114.40 (15)	C27—C22—C9	123.38 (16)
C6—C9—C22	110.16 (15)	C22—C23—C24	122.36 (19)
C10—C9—H9	108.0	C22—C23—H23	118.8
C6—C9—H9	108.0	C24—C23—H23	118.8
C22—C9—H9	108.0	C25—C24—C23	119.76 (19)
C15—C10—C11	121.21 (17)	C25—C24—H24	120.1
C15—C10—C9	121.43 (17)	C23—C24—H24	120.1
C11—C10—C9	117.35 (16)	O3—C25—C24	125.15 (19)
O2—C11—C10	121.36 (17)	O3—C25—C26	115.88 (19)
O2—C11—C12	121.14 (18)	C24—C25—C26	119.0 (2)
C10—C11—C12	117.48 (17)	C27—C26—C25	120.4 (2)
C11—C12—C13	112.51 (17)	C27—C26—H26	119.8
C11—C12—H12A	109.1	C25—C26—H26	119.8
C13—C12—H12A	109.1	C26—C27—C22	121.77 (19)
C11—C12—H12B	109.1	C26—C27—H27	119.1
C13—C12—H12B	109.1	C22—C27—H27	119.1
H12A—C12—H12B	107.8	O3—C28—H28A	109.5
C12—C13—C17	110.69 (19)	O3—C28—H28B	109.5
C12—C13—C14	107.84 (17)	H28A—C28—H28B	109.5
C17—C13—C14	110.58 (18)	O3—C28—H28C	109.5
C12—C13—C16	109.66 (17)	H28A—C28—H28C	109.5
C17—C13—C16	109.12 (19)	H28B—C28—H28C	109.5
C14—C13—C16	108.92 (18)		
			
C15—N1—C1—C6	−12.1 (3)	C11—C12—C13—C14	56.7 (2)
C18—N1—C1—C6	172.94 (18)	C11—C12—C13—C16	175.2 (2)
C15—N1—C1—C2	166.47 (18)	C12—C13—C14—C15	−48.6 (2)
C18—N1—C1—C2	−8.4 (3)	C17—C13—C14—C15	72.5 (2)
C6—C1—C2—C3	−10.9 (3)	C16—C13—C14—C15	−167.55 (18)
N1—C1—C2—C3	170.49 (18)	C11—C10—C15—N1	−172.16 (17)
C1—C2—C3—C4	44.3 (3)	C9—C10—C15—N1	6.7 (3)
C1—C2—C3—C8	−76.6 (3)	C11—C10—C15—C14	5.2 (3)
C1—C2—C3—C7	163.1 (2)	C9—C10—C15—C14	−175.93 (17)
C2—C3—C4—C5	−56.0 (3)	C1—N1—C15—C10	16.1 (3)
C8—C3—C4—C5	64.9 (3)	C18—N1—C15—C10	−168.99 (18)
C7—C3—C4—C5	−173.9 (2)	C1—N1—C15—C14	−161.44 (16)
C3—C4—C5—O1	−147.1 (2)	C18—N1—C15—C14	13.5 (3)
C3—C4—C5—C6	34.8 (3)	C13—C14—C15—C10	19.1 (3)
N1—C1—C6—C5	165.40 (17)	C13—C14—C15—N1	−163.47 (17)
C2—C1—C6—C5	−13.2 (3)	C15—N1—C18—C19	83.4 (2)
N1—C1—C6—C9	−14.3 (3)	C1—N1—C18—C19	−101.7 (2)
C2—C1—C6—C9	167.16 (17)	C21—N2—C19—C18	−87.4 (2)
O1—C5—C6—C1	−177.22 (19)	C20—N2—C19—C18	147.7 (2)
C4—C5—C6—C1	0.8 (3)	N1—C18—C19—N2	171.31 (17)
O1—C5—C6—C9	2.5 (3)	C10—C9—C22—C23	147.68 (18)
C4—C5—C6—C9	−179.53 (18)	C6—C9—C22—C23	−90.4 (2)
C1—C6—C9—C10	33.0 (2)	C10—C9—C22—C27	−35.8 (3)
C5—C6—C9—C10	−146.74 (17)	C6—C9—C22—C27	86.2 (2)
C1—C6—C9—C22	−92.7 (2)	C27—C22—C23—C24	−0.3 (3)
C5—C6—C9—C22	87.62 (19)	C9—C22—C23—C24	176.46 (19)
C6—C9—C10—C15	−29.1 (2)	C22—C23—C24—C25	−0.6 (3)
C22—C9—C10—C15	94.0 (2)	C28—O3—C25—C24	2.7 (3)
C6—C9—C10—C11	149.79 (16)	C28—O3—C25—C26	−177.5 (2)
C22—C9—C10—C11	−87.1 (2)	C23—C24—C25—O3	−179.0 (2)
C15—C10—C11—O2	−178.41 (19)	C23—C24—C25—C26	1.2 (3)
C9—C10—C11—O2	2.7 (3)	O3—C25—C26—C27	179.3 (2)
C15—C10—C11—C12	3.3 (3)	C24—C25—C26—C27	−0.8 (4)
C9—C10—C11—C12	−175.59 (17)	C25—C26—C27—C22	−0.1 (4)
O2—C11—C12—C13	146.1 (2)	C23—C22—C27—C26	0.6 (3)
C10—C11—C12—C13	−35.6 (3)	C9—C22—C27—C26	−176.0 (2)
C11—C12—C13—C17	−64.3 (2)		

### Hydrogen-bond geometry (Å, °)

**Table:**

*D*—H···*A*	*D*—H	H···*A*	*D*···*A*	*D*—H···*A*
C14—H14B···O1^i^	0.97	2.51	3.368 (2)	147

Symmetry codes: (i) *x*−1/2, −*y*+1/2, *z*+1/2.
